# Delayed diagnosis of scaphocephaly after 1 year of age is associated with elevated intracranial pressure

**DOI:** 10.3389/fped.2026.1928890

**Published:** 2026-10-27

**Authors:** H. Luz, A. Bertrand, M. Luxenhofer, P. Esslinger, M. Lehner, D.S. Wendling-Keim

**Affiliations:** 1 Department of Pediatric Surgery, Children's Hospital of Central Switzerland, Lucerne, Switzerland; 2 Department of Neurology, Rechts der Isar Hospital, TUM University Hospital, Munich, Germany; 3 Department of Pediatric Surgery, Dr. von Hauner Children's Hospital, Munich, Germany

**Keywords:** cranial vault remodeling, craniosynostosis, ICP monitoring, modified Pi-plasty, scaphocephaly

## Abstract

**Purpose:**

Delayed presentation of scaphocephaly remains clinically challenging, particularly regarding the risk of elevated intracranial pressure (ICP) and the management of late surgical intervention. This study evaluated the prevalence of elevated ICP in children with delayed diagnosis and the effect of decompressive cranial vault surgery.

**Methods:**

A retrospective single-center analysis included five patients with delayed diagnosis (May 2022–January 2024) and three infants treated early as a reference group. Clinical characteristics, cranial index (CI), ophthalmologic findings, and intraoperative ICP were analyzed. All delayed cases underwent modified Pi-plasty with systematic ICP monitoring.

**Results:**

The delayed group had a mean CI of 0.64, compared with 0.68 in the early group. Papillary edema was present in two delayed cases and absent in controls. Mean opening ICP in delayed patients was 24 mmHg (range 16–36), significantly higher than 5 mmHg in early-treated patients (*p* < 0.01). Following surgery, ICP decreased to a mean of 7.5 mmHg.

**Conclusion:**

All patients with delayed diagnosis and treatment of scaphocephaly showed elevated ICP in this study. Decompressive cranial vault surgery effectively normalized ICP, highlighting the importance of early diagnosis and timely intervention to reduce the risk of intracranial hypertension.

## Introduction

Craniosynostosis is characterized by premature fusion of one or multiple cranial sutures. Skull growth is restricted perpendicular to the affected suture with compensatory expansion along the remaining patent sutures (1). This growth pattern, known as Virchow's law, leads to characteristic cranial deformities and may impair normal development of the visceral and neurocranium. Craniosynostosis affects about 1 in2000–2,500 live births and can occur either as part of a genetic syndrome or as an isolated anomaly (2). The most common form of isolated single-suture craniosynostosis is scaphocephaly, caused by a prematurely fused sagittal suture (3). Affected children present with characteristic anterior–posterior lengthening and compensatory frontal and occipital bossing (4). The severity of skull deformity can be clinically assessed using the cranial index (CI). The mean CI for healthy children under the age of 3 years is 0.81 ± 0.7 (5, 6).

Beyond cranial shape abnormalities, craniosynostosis may affect various systems; the most common are otorhinolaryngologic and ophthalmologic manifestations. These latter ones include strabismus, amblyopia, refractive error, and papillary edema leading to atrophy (7–9). Although these symptoms occur more frequently in syndromic cases such as Crouzon and Apert syndromes, approximately 7%–44% of patients with scaphocephaly are at risk of developing elevated ICP, with subsequent ophthalmologic complications, including blindness (7, 10–12).

This study aims to investigate patients with a delayed diagnosis of scaphocephaly focusing on the prevalence of elevated ICP in this cohort, as well as the effect of decompressive cranial vault surgery on ICP reduction. These findings aim to add to the ongoing discussion regarding indications, timing, and surgical management strategies in patients presenting beyond infancy.

## Materials and methods

We performed a single-center, retrospective study including five patients with delayed diagnosis of scaphocephaly who presented to our institution between May 2022 and January 2024. The inclusion criteria encompassed all patients presenting within the specified timeframe with isolated single-suture scaphocephaly diagnosed after age 1 year. Patients with syndromic scaphocephaly and those diagnosed and surgically treated as infants were excluded.

Data collection involved a meticulous review of medical records, imaging studies, and clinical assessments conducted during the visits of patients. The variables assessed included demographic information, clinical presentation, diagnostic imaging results (such as x-rays and MRI scans), cranial index measurements, the presence of papillary edema, and intraoperative documentation, including ICP measurements.

All patients underwent a standardized preoperative ophthalmologic evaluation by pediatric ophthalmologists, including fundoscopy to assess papillary edema. All patients were operated on under general anesthesia and complete neuromuscular blockade. Anesthesia was performed by the same team of anesthesiologists for all patients included in this study.

Patients in the delayed-treatment cohort underwent modified Pi-plasty according to the technique originally described by Jane et al. and subsequently modified by other groups (13–15).

Intraoperative ICP measurements were routinely achieved as intraparenchymal opening pressures just before starting the craniectomy and as closing pressures just before completing the reconstruction.

ICP monitoring was performed using the Raumedic Neurovent-PX® ICP measuring probe. In all cases, including osteoclastic craniectomy and modified Pi-plasty, the first burr hole was performed in the right frontal region just in front of the coronal suture. After bipolarization of the dura, the dura was incised, the measuring probe was inserted 2 cm into the parenchyma, and the probe was connected to the anesthesia monitoring system. The probe was fixated in the burr hole using bone wax and, if possible, left in place until the reconstruction was finished. In two cases—one in the study group and one in the control group—the probe dislocated during reconstruction and was not reinserted.

Intracranial pressure (ICP) ≥15 mmHg under general anesthesia was defined *a priori* as pathologically elevated. Three infants diagnosed and treated for scaphocephaly during this time by osteoclastic craniectomy as infants were used as a reference group. The surgical technique was as previously described by Lehner et al. (16).

Statistical analysis was performed using an unpaired t-test and *post-hoc* analysis, as indicated, using GraphPad Prism version 7.00 (GraphPad Software, La Jolla, CA, USA), assuming normal distribution of the values and equality of variances. An alpha level of <0.05 was considered statistically significant. The study adhered to ethical guidelines of our institution, and compliance to privacy regulations was ensured.

## Results

### Patient demographics and characteristics

Over a period of 2 years, we analyzed five cases with delayed diagnosis of scaphocephaly. Age at surgery ranged from 19 to 62 months. This cohort consisted of three males and two females, all displaying the characteristic cranial morphology associated with scaphocephaly.

For comparison, a control group of three infants who underwent standard osteoclastic craniectomy for scaphocephaly during the same study period was included. These infants, two boys and one girl, were operated on at the ages of 8, 6, and 5 months, respectively. As expected, the age of the control group was significantly lower than that of the delayed-treatment group (*p* < 0.05). The age at surgery for all patients is shown in Figure 1.

**Figure 1:**
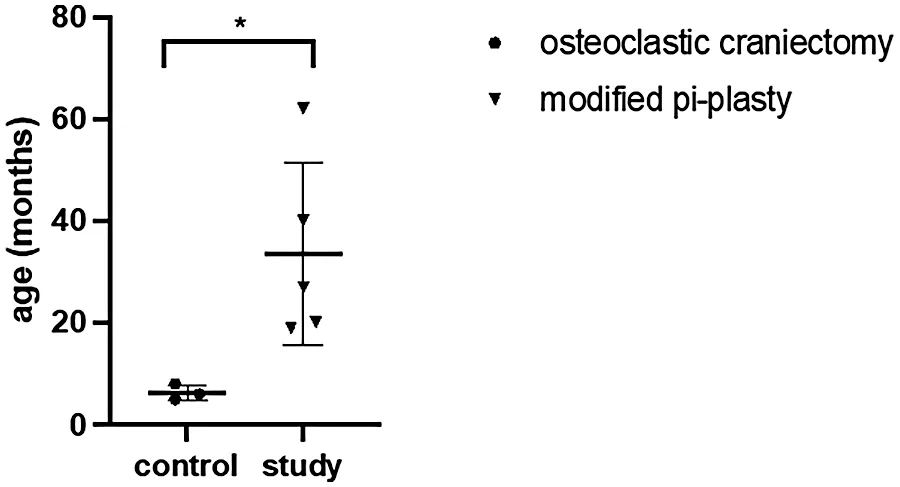
Age of patients undergoing osteoclastic craniectomy or modified pi-plasty in the control group and the study group.

### Papillary edema and associated conditions

All patients were assessed for papillary edema by pediatric ophthalmologists. Two patients in the delayed-treatment group demonstrated papillary edema at presentation, whereas no evidence of papillary edema was observed in the remaining three patients. No cases of papillary edema were detected in the control group. Incidentally, one patient in the late treatment group had a concurrent diagnosis of Alagille syndrome, and another was found to have a Chiari type 1 malformation. No papillary edema was present in the patient with Chiari type 1 malformation.

### Cranial index

In the delayed-treatment group, the mean CI was 0.64 (range, 0.60; 0.65), with no patient showing a CI within the normal range.

In the control group, the mean CI was 0.68 (range, 0.67–0.69). There was no significant difference in the preoperative CI between the two groups (*p* = 0.14). The preoperative cranial index values are shown in Figure 2.

**Figure 2:**
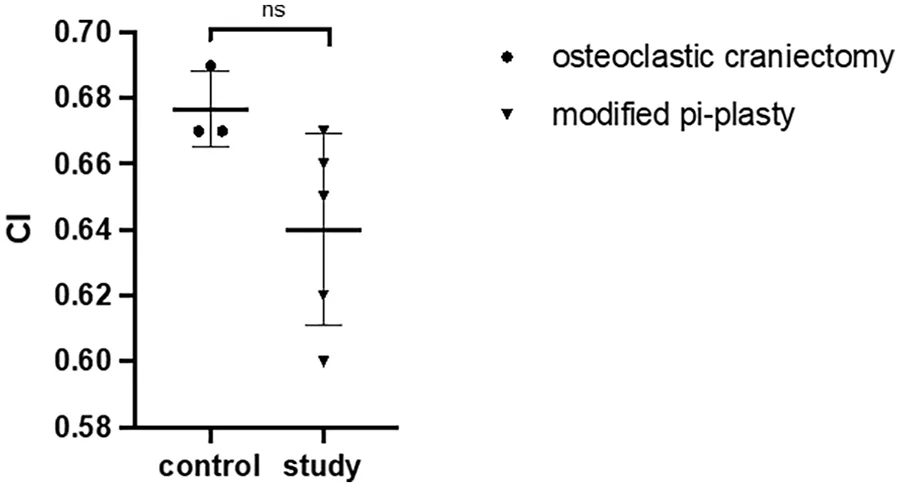
Demonstrates the cranial vault index of the patients in the control group and study group after osteoclastic craniectomy or modified pi-plasty.

### Clinical assessment and indication for surgery

To highlight the clinical presentation and rationale for surgical intervention in patients with delayed diagnosis of scaphocephaly, individual case summaries are provided below. An overview is presented in Table 1.

Case 1: A 5-year-old boy was referred to our clinic for persistent papillary edema of unknown origin, present for more than 2 years. Previous lumbar puncture by pediatric neurologists had not shown elevated ICP. Clinical examination revealed a characteristic scaphocephalic cranial morphology with a CI of 0.67. Neurodevelopment was age-appropriate. The x-ray showed a copper-beaten skull suggestive of chronic intracranial hypertension, and an indication for surgery was given after securing the diagnosis of scaphocephaly and suspected ICP elevation.
Case 2: An 18-month-old girl was referred to our clinic by her pediatrician with a scaphocephalic head shape after repeated parental concerns regarding her head shape. Although the child did not demonstrate papillary edema, the x-ray showed a significantly copper-beaten skull as an indirect sign of elevated ICP. The CI at presentation was 0.66. Neurodevelopment was normal for age. An indication for surgery was given because of suspected ICP elevation.
Case 3: An 18-month-old boy was referred to our clinic by the primary care pediatrician for an abnormal head shape. The CI at presentation was 0.6. Clinical examination confirmed scaphocephaly and x-ray revealed a copper-beaten skull. Bilateral papillary edema was present. MRI ruled out additional intracranial pathologies, and an indication for surgery was given. Neurologically, the child had been developing well without any signs of delay.
Case 4: A 3-year-old girl was introduced to us by her parents because of the same abnormal head shape as her brother, who had recently been diagnosed with scaphocephaly. She demonstrated normal neurodevelopment. The CI at presentation was 0.65. Although ophthalmological examination showed no papillary edema, an x-ray showed a copper-beaten skull. An MRI revealed a Chiari type 1 malformation without associated hydrocephalus or syrinx. An indication for surgery was given due to the copper-beaten skull and the Chiari malformation to relieve any supratentorial pressure.
Case 5: This patient had been evaluated in infancy for scaphocephaly, which was already suspected prenatally. The boy presented with a scaphocephalic head shape, but his parents refused surgery during his infancy. By the age of 8 months, the x-ray revealed a copper-beaten skull, whereas ophthalmologists did not detect papillary edema. The boy developed age-appropriately. Repeated ophthalmological assessments performed every 6 months did not demonstrate papillary edema. After a more pronounced scaphocephalic head shape with a CI of 0.62, the parents consented to surgery at the age of 27 months.

**Table 1:** Case summary of clinical data, examinations performed, and intraoperative data.

Clinical characteristics	Gender	Age at surgery (m)	CI	Papillary edema	X-ray: copper-beaten skull	MRI	CT	Opening pressure (mmHg)
Case 1	Male	62	0.67	Yes	Yes	Papillary edema, rule out tumor	Yes, preoperative planning	16
Case 2	Female	19	0.66	No	Yes		Yes, preoperative planning	25
Case 3	Male	20	0.6	Yes	Yes	Papillary edema, rule out tumor		36
Case 4	Female	40	0.65	No	Yes	Late diagnosis, revealed Chiari 1 malformation		18
Case 5	Male	27	0.62	No	Yes			25

### Intracranial pressure monitoring

All five patients in the delayed-treatment group underwent modified Pi-plasty procedures with systematic intraoperative ICP monitoring. The mean opening pressure was 24 mmHg (SD 7.8; range, 16–36 mmHg), which significantly decreased to a mean closing ICP of 7.5 mmHg (SD 1.7) following cranial vault expansion.

In comparison, in the control group, the mean opening pressure was 5 mmHg (SD 1), which was significantly lower than that observed in the delayed-treatment cohort (*p* < 0.01). Notably, all patients in the delayed-treatment cohort had pathologically elevated opening ICP (≥15 mmHg), whereas none in the reference cohort exhibited elevated ICP. All measurements were taken under general anesthesia. The ICP measurements are shown in Figure 3.

**Figure 3:**
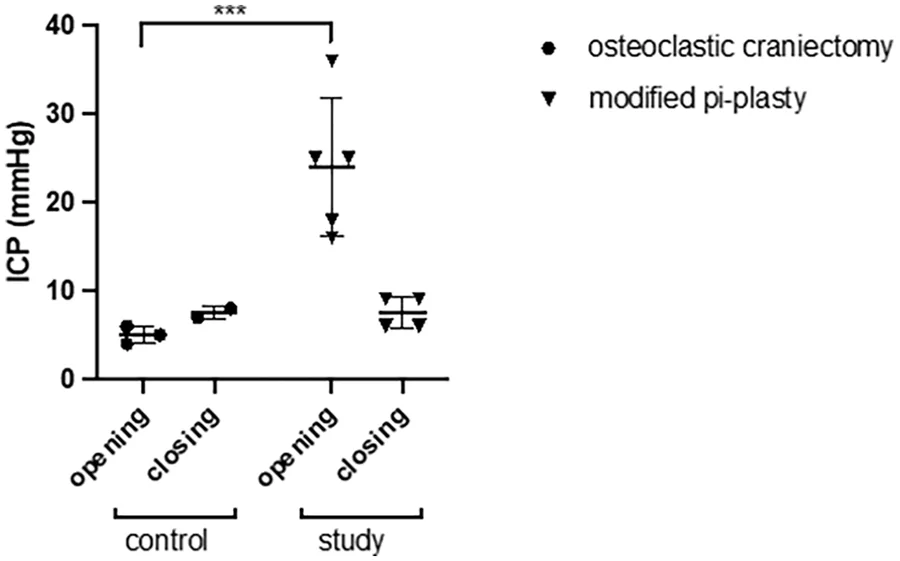
Compares the opening and closing ICP of both treatment groups after osteoclastic craniectomy or modified pi-plasty.

### Follow-up

All children are still in active follow-up in our clinic. All participants remain under follow-up through 2026. At the latest follow-up, all patients demonstrated stable clinical outcomes without recurrent papillary edema or neurological deterioration, and no patient required reoperation. A summary of the follow-up data is shown in Table 2.

Case 1: The papillary edema had been in complete remission 3 months after surgery and has not recurred during 3 years of follow-up. The postoperative course has otherwise been uneventful. Clinically and neurologically, the boy is developing age-appropriately.
Case 2: The initial postoperative course was uneventful. The girl developed a minor wound dehiscence about 3 weeks postoperatively, which healed without the need for revision surgery. One year postoperatively, no papillary edema was present.
Case 3: The postoperative course was uneventful. Although the boy had been developing age-appropriately preoperatively, he demonstrated notable gains in overall development and language development during the first 3 months after surgery. The papillary edema resolved completely and has not recurred 2 years postoperatively.
Case 4: The postoperative course was uneventful. The Chiari malformation remained unchanged, with no signs of hydrocephalus at 6 and 12 months postoperatively. There has been no evidence of papillary edema at 6 and 12 months postoperatively.
Case 5: The postoperative course has been uneventful. There is no sign of papillary edema 12 months postoperatively. The neurodevelopment has remained age-appropriate.

**Table 2:** Case summary of the clinical follow-up.

Clinical follow up	Intracranial pressure (mmHg)		Papillary edema		Complications	Clinical observations
	Opening pressure	Closing pressure	Preoperative	Postoperative		
Case 1	16		Yes	Complete remission	None	
Case 2	25	9	No		Wound dehiscence, no revision surgery	Leap in general and language development within 3 months postoperatively
Case 3	36	9	Yes	Complete remission	None	Chiari malformation unchanged
Case 4	18	6	No		None	
Case 5	25	9	No		None	

## Discussion

Over the course of 2 years, we treated five children with scaphocephaly at our institution who were diagnosed and managed past 12 months of age. Whether and how children older than 1 year should be treated remains a subject of ongoing discussion (17).

Although the prevalence of ICP elevation is believed to be much higher in multi-sutured and syndromic craniosynostosis cases at roughly 45%, depending on current literature, approximately 7%–44% of scaphocephaly patients are estimated to be affected, too (11, 12, 18–23). However, determining the exact prevalence of elevated ICP is a formidable challenge, as evidenced by considerable disparities in prevalence estimates across various epidemiological studies. This complexity arises from heightened difficulties in data acquisition due to the low incidence. In addition, variations in study design play a crucial role, encompassing differences in measurement methods, the demographic composition of study populations, particularly with respect to age, and the establishment of normative value ranges.

Interestingly, previous studies suggest that the prevalence of ICP elevation might increase over time if single-sutured craniosynostosis is not operated on and might thus be higher in older children. Arnaud et al. reported a higher prevalence in patients older than 1 year than in patients below 1 year (26% vs. 7%) (21). Accordingly, it was shown that patients with single-suture craniosynostosis benefit from early surgery, as they have a lower postoperative risk of ICP elevation than older children (24). Tuite et al. found in their study population (*n* = 122) that children with papillary edema and ICP elevation (*n* = 13) were older than those without (5.9 vs. 1.9 years) (20). Also, Delattre et al. examined non-operated adult scaphocephalic skulls and found radiologic signs of elevated ICP, such as copper-beaten skull (25).

In our study, although only two of our eight children had papillary edema, all five in the late treatment group had significantly elevated ICP at the start of their operation (mean 24 mmHg, SD 7.8). In sharp contrast, none of the children in the control group showed elevated ICP (mean 5 mmHg, SD 1).

This also raises the question of whether we can adequately recognize and monitor signs of intracranial pressure in children.

The prevalence of ICP elevation in scaphocephaly cases in general underscores the importance of vigilant monitoring, especially considering the potential age-dependent increase in incidence, as indicated by various studies (12, 20, 21, 24–29).

As increased ICP is associated with severe complications, the early detection of affected patients is essential to facilitate treatment. Some studies suggest that earlier surgery may be associated with more favorable neurocognitive outcomes. Patel et al. demonstrated that children with craniosynostosis who underwent surgery before age 6 months had significantly better neurological outcomes. Compared with children who were operated on between the ages of 6–12 months and after 12 months, those who received early surgery had higher full-scale and verbal IQs (30). Bellew et al. found higher full-scale IQ at 10 years among children treated before 7 months. However, Starr et al. found no strong association between age at repair and neurodevelopmental performance at 3 years. A systematic review by Mandela et al. found that 5 of 10 studies supported a benefit of earlier surgery, whereas five did not. This underscores the ongoing controversy surrounding this issue and the need for further research (30) (31) (32) (33). However, the diagnostic evaluation of raised ICP poses a challenge to the practitioner. The gold standard involves an intracranial measurement with procedure-associated risks. The ideal surrogate for ICP measurement would be non-invasive, safe, objective, and highly sensitive.

A commonly used indicator for ICP elevation is papillary edema, the swelling of optic nerve axons. Accordingly, given the prevalence of ICP elevation, papillary edema also affects 9%–15% of scaphocephaly patients (34, 35). Fundoscopy is the classic ophthalmologic examination for detecting papillary edema. However, in our study cohort, only two of the five children had papillary edema, whereas all patients demonstrated elevated ICP. Accordingly, there is broad consensus in the literature that this method is an appropriate tool to screen for ICP elevation but not to rule it out, as the negative predictive value is low (24, 36).

Optical coherence tomography is a newer, suitable method for examining children's peripapillary retinal structures and detecting papillary edema. Norm ranges have been established, and the successful detection of papillary edema has already been demonstrated in children (37, 38). Data suggest that optical coherence tomography (OCT) detects abnormalities earlier than fundoscopy but is only feasible in children older than 3 years (7, 39). Accordingly, a prospective cohort study showed that OCT detected more abnormalities than fundoscopy (15% vs. 4%) in a group of single-suture patients older than 3 years with clinical signs of elevated ICP. When comparing OCT results with intracranial ICP measurements, a sensitivity of 89% and a specificity of 62% were reported. This study was performed with children affected by craniosynostosis or hydrocephalus; an elevated ICP was defined as >15 mmHg (40). Also, they established reference values and suggested a standard screening combination of OCT + fundoscopy (39).

In summary, OCT detects papillary edema earlier and more objectively than fundoscopy but is only feasible in older children.

An increase in the optic nerve sheath diameter indicates papillary edema of the optic nerve and can be measured on trans-bulbar sonography, CT, or MRI scans. This method seems promising because it is feasible in patients younger than 1 year (41). In adults, its diagnostic accuracy with a sensitivity of 90% and specificity of 85% has already been established (42). However, data from pediatric patients seem less promising, as their diagnostic reliability is widely discussed in the literature (43).

Another challenge of ICP diagnosis concerns the fact that there is no final consensus on normal ranges of ICP in children. A meta-analysis of pediatric TBI research concluded that an ICP threshold of 20 mmHg is generally considered a treatment indication (44). However, deep anesthesia lowers the ICP. To date, there is no reliable, age-dependent reference threshold for normal ICP ranges in infants and children under anesthesia. In a recent study involving infants with craniosynostosis, Makoshi et al. used an ICP threshold of >10 mm Hg during general anesthesia to classify elevated intracranial pressure (45). Therefore, the threshold of 15 mm Hg was selected based on prior pediatric craniosynostosis literature and was intended as a classification threshold rather than a therapeutic trigger. The threshold used is a commonly used, disease-specific classification threshold supported by prior craniosynostosis literature; however, we acknowledge that no universal pediatric cutoff exists (40, 46).

Further, the control group was small because only three eligible patients with complete ICP data were available during the study period. Consequently, comparisons with the control group should be regarded as exploratory, and future studies are needed to confirm the findings of this pilot study. Nevertheless, we note that the measurements across the three cases were highly consistent. This consistency is relevant because it means that the three cases do not constitute an overly heterogeneous control group; the comparison group is internally coherent rather than a loose collection of divergent observations. The indication for surgery can be made for two reasons, or a combination of them (10, 47, 48): ICP elevation with possible neurologic or ophthalmologic symptoms, and severe skull deformation with possible functional, psychosocial, and aesthetic impairments.

It is agreed that immediate surgical treatment is required if ICP elevation is detected (49). Back in 1988, Kaiser showed that surgical treatment can reduce neurological symptoms of elevated ICP (50). Evidence of actual ICP lowering by surgery was brought in 2018. There, they showed a significant difference between pre- and postoperative ICP using the gold standard method of intracranial monitoring (24). Recently, Makoshi et al. showed a similar effect in patients with strip craniectomy (45).

Still, it is also known that the later the surgery is performed, the lower the risk of reoperation, as the bone reconstruction is supposedly more stable and the recurrence of ICP elevation is unlikely (51–54). In a series of 217 non-syndromic cases, 6.9–14.6% showed postoperative ICP elevation. The authors identified the surgical procedure and age as factors but could not determine which was more significant. Notably, they found that postoperative ICP elevation was more frequent in patients with prior ICP elevation (53). The possibility of ICP re-elevation after surgical correction indicates the need for close follow-up postoperative examinations (54).

This pilot study has several limitations. Its retrospective design introduces the possibility of selection bias, incomplete documentation, and unmeasured confounding. The small sample size, particularly the control group, which comprised only three eligible patients with complete ICP data, limits statistical analysis. Accordingly, the results should be interpreted as preliminary findings intended to inform the design of larger prospective studies. Also, intraoperative ICP may fluctuate with anesthesia, ventilation, head position, surgical manipulation, or probe displacement. Although the probe was secured in the burr hole, it became dislodged during reconstruction in two patients and was not reinserted. Moreover, thresholds derived from prolonged monitoring may not be directly applicable to intraoperative measurements. Finally, although 15 mmHg is commonly used as a disease-specific classification threshold in the pediatric craniosynostosis literature, no generally accepted pediatric ICP cutoff exists across age groups. Larger prospective studies using standardized measurement protocols are needed to validate these preliminary findings.

## Conclusion

Given the significant prevalence and potential long-term consequences of elevated ICP in patients with scaphocephaly, particularly in those diagnosed and treated beyond infancy, and the demonstrated benefits of early surgical intervention in improving both neurological outcomes and reducing ICP, we advocate for operative treatment once a clear indication is established. Early surgical correction not only lowers the risk of elevated ICP but may also mitigate aesthetic, psychosocial, and functional impairments.

## Data Availability

The raw data supporting the conclusions of this article will be made available by the authors, without undue reservation.
